# A crucial role of miR-155 in the pathomechanism of acute kidney injury

**DOI:** 10.3389/fphar.2025.1570000

**Published:** 2025-04-16

**Authors:** Hui Wang, Jian Liao, Wei Wang, Jianhua Zhang

**Affiliations:** ^1^ Department of Urology, The First People’s Hospital of Linhai, Linhai, Zhejiang, China; ^2^ Department of Nephrology, Jiaxing Hospital of Traditional Chinese Medicine, Jiaxing, China; ^3^ Department of Urology, Tiantai People’s Hospital of Zhejiang Province (Tiantai Branch of Zhejiang Provincial People’s Hospital), Hangzhou Medical College, Taizhou, Zhejiang, China

**Keywords:** AKI, mir-155, mechanism, therapeutic targets, apoptosis, inflammation

## Abstract

Acute kidney injury (AKI) is one of the nonnegligible causes of mortality worldwide. It is important to understand the underlying molecular mechanism of AKI to effective therapeutic targets. miR-155 has been found to play a pivotal role in the development of AKI, while a comprehensive review on this topic is currently still lacking. Based on this review, we found that miR-155and is strongly correlated with the pathophysiological development of AKI by modulating cell apoptosis, inflammation, and proliferation. Mechanistically, miR-155 exerts a promoting function in multiple types of AKI by regulating multiple proteins or signaling pathways, such as SOCS-1, ERRFI1, SOCS-1, TRF1, CDK12, and TCF4/Wnt/β-catenin pathway. The inhibition of miR-155 has a renoprotective effect in drug- or substance-induced AKI. Therefore, drugs or biological compounds targeted by miR-155 and its pathways may recover the process of AKI by altering apoptosis, inflammation, and pyroptosis. A miRNA nanocarrier system that has already been developed could offer a novel approach to treat AKI, providing a direction for future research. Further large-scale studies are necessary to elucidate the clinical significance of miR-155 as a potential therapeutic target for multiple types of AKI.

## Introduction

Acute kidney injury (AKI) is an abrupt loss of kidney function, characterized by the retention of nitrogenous and a decline in glomerular filtration rate (GFR) (Birkelo et al., 2022). Due to a lack of specificity, the Acute Dialysis Quality Initiative (ADQI) has classified AKI based on the Risk, Injury, Failure, Loss of kidney function, and End Stage Renal Disease (RIFLE) in 2004 (Wlodzimirow et al., 2012). Following that, a modification of the RIFLE criteria was proposed by the Acute Kidney Injury Network (AKIN) (Hong et al., 2023). The most recent guideline, introduced by the Kidney Disease Improving Global Outcomes (KDIGO) workgroup in 2012, defines AKI as an increase of creatinine of 0.3 mg/dL within 48 h or an increase of 50% in 7 days (Haredasht et al., 2023). AKI, a common clinical disease, is the leading cause of mortality worldwide (Lu et al., 2023; de Ponte et al., 2021). The global burden of AKI-related mortality rate far exceeded those of diabetes mellitus, breast cancer, heart failure or and prostate carcinoma (Alassaf and Attia, 2023). Despite advances in treatment AKI in the last 50 years, the mortality rates remaining high (Kellum et al., 2021). It was reported that overall mortality rate of patients with AKI was 23% and was 49.4% in those requiring kidney replacement therapy (Susantitaphong et al., 2013). Risk factors of AKI include socioeconomic, cultural and environmental factors, as well as factors related to acute exposures, the process of care and patients themselves (Mehta et al., 2015). Patient-related factors include hypotension, hypoxia, anaemia, chronic kidney, diabetes, severe sepsis, and use of nephrotoxic drugs. Further important risk factors of AKI are severe trauma, old age, acute organ failures, delayed graft function upon kidney transplantation and major surgeries (including cardiac surgery) (Mehta et al., 2016). Currently, avoidance of nephrotoxins and hemodynamic and fluid status optimization are the main methods for treating AKI(Pickkers et al., 2021). However, owing to complex pathophysiology, variable clinical presentation and heterogeneous syndromes, specific pharmacologic therapies are hindered especially in high-risk situations (Zhao J. et al., 2022). Therefore, there is an urgent need to uncover novel potential therapeutic targets for AKI. Recently, a growing number of studies showed that microRNAs (miRNAs) might play essential roles in AKI.

MiRNAs, part of the epigenome, are endogenous short and non-coding RNA molecules (containing∼22 nucleotides) that play important roles in regulating the expression of the target gene at the post-transcriptional level by promoting the degradation of mRNA through binding the 3′untranslated region of gene mRNA (Ferragut et al., 2021). MiRNAs are transcribed by RNA-polymerase II and then processed sequentially by the enzymes drosha ribonuclease III (DROSHA) and dicer 1 ribonuclease III (DICER) (Sun et al., 2022). A single miRNA can modulate hundreds of mRNAs and alter the expression of many genes (Garcia-Blanco et al., 2022). MiRNAs exert complex and crucial functions in many biological processes, including cell proliferation, apoptosis, diseases, and development (Li X. et al., 2021; Shafat et al., 2022; Cui et al., 2022). Recently, miRNAs were shown to play roles the pathogenesis of spinal cord injury, traumatic brain injury and AKI (Hu et al., 2022; Tod et al., 2020; Wang F. et al., 2022). In recent years, researchers have been paying more and more attention to the role of miR-155 in AKI. However, systematic reviews addressing the roles of miR-155 in AKI are lacking. In this paper, we present a first attempt to summarize the current knowledge about miR-155 in the progress of AKI. The objective of this review is to provide readers with an overview of this important topic that prompts the clinical application of miRNA-155-based therapeutics.

### Overview of miRNA-155

**FIGURE 1:**
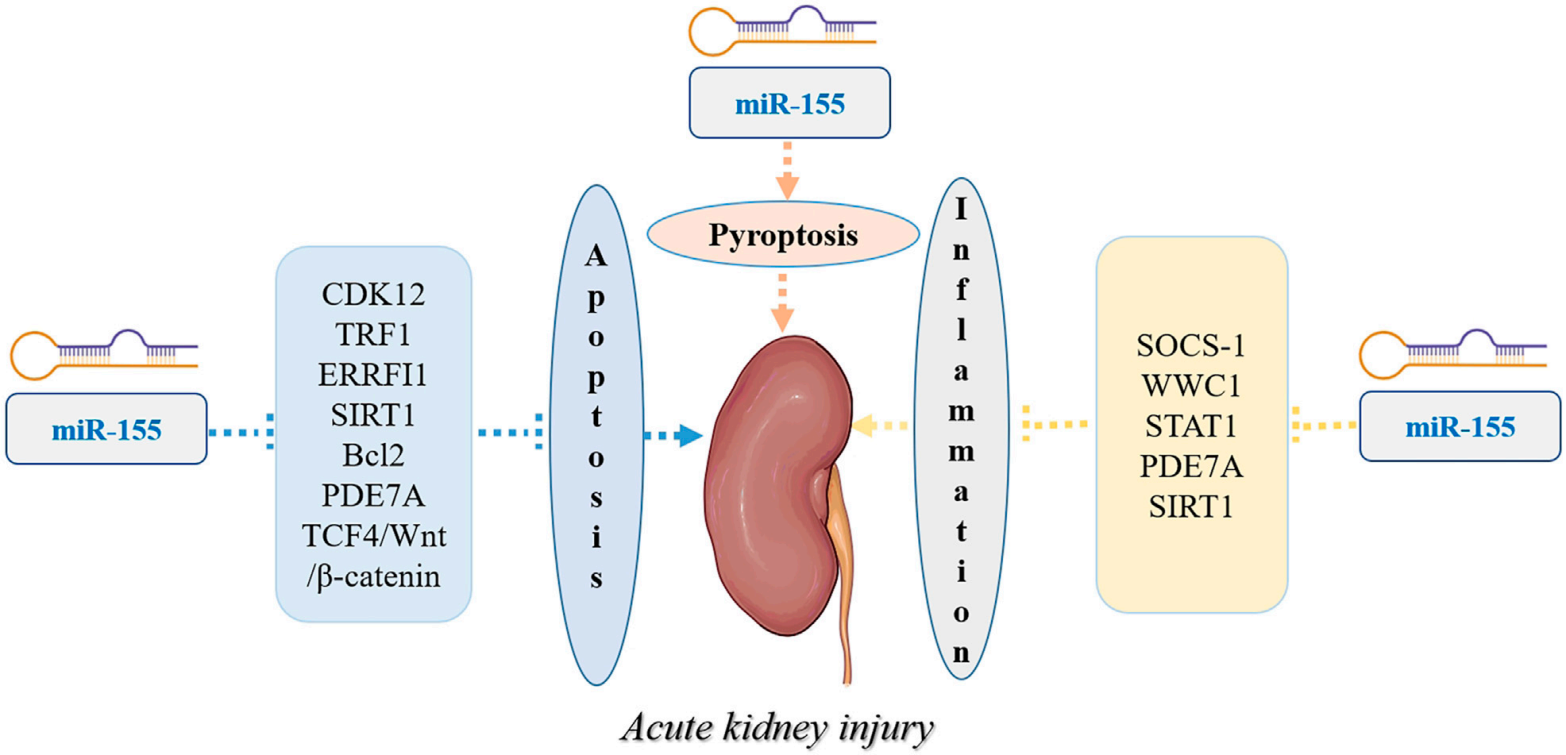
Biogenesis, function, and regulation of miR-155.

MiR-155 is encoded by the miR-155 gene, which is located on chromosome 21 in humans (Perez-Villarreal et al., 2022). The miR-155 gene is transcribed by RNA polymerase II into a long primary transcript known as primary miR-155 (pri-miR-155) (Lin et al., 2022). Pri-miR-155 undergoes several processing steps to generate the mature miRNA molecule (Lin et al., 2022). The miR-155 host gene produces two different miRNA strands, miR-155-3p and miR-155-5p, where miR-155-5p is the functional miR-155 form (Mycko et al., 2015). The mature miR-155 is approximately 22 nucleotides in length (Mahesh and Biswas, 2019). Like other microRNAs, it has a characteristic stem-loop structure (Liu et al., 2016). The mature miR-155 sequence is contained within one arm of the stem-loop structure, with the complementary sequence forming the other arm (Liu et al., 2016). This stem-loop structure is essential for the processing and stability of miR-155 (Liu et al., 2016). Mature miR-155 functions as a post-transcriptional regulator of gene expression by binding to the 3′untranslated region (UTR) of target messenger RNA (mRNA) molecules (Jankauskas et al., 2021). This binding leads to mRNA degradation or translational repression, depending on the degree of complementarity between miR-155 and its target mRNA (Jankauskas et al., 2021). Figure 1 shows the Biogenesis, function, and regulation of miR-155.

Over the past decade, miR-155 has become a hot research spot. Multiple studies showed that miR-155 played essential roles in various biological processes such as cell apoptosis, proliferation, migration and differentiation, and was significantly differentially expressed in a wide range of tumors, inflammatory diseases and lung injury (Xu et al., 2023; Ji et al., 2022; Gulei et al., 2019; Xu W. D. et al., 2022). Sepsis and drugs are also frequent causes of AKI. A recent study showed that the expression of miR-155 in the septic non-AKI was significantly lower than that in the septic AKI(Fan et al., 2023). Therefore, miR-155 may show a protective or aggravating effect on the progress of AKI by regulating different target proteins or signaling pathways.

### Literature search

Four databases, Pubmed, Scopus, EBSCOhost, MEDLINE were searched to maximally identify eligible studies, which were summarized using rational systematic review methods. The keyword search strategy in PubMed was: (((((Acute Kidney Failure) OR (Acute Renal Failure)) OR (Acute Kidney injury)) OR (Acute renal injury))) AND (((((((miR-155) OR (miR155)) OR (miR-155, microRNA)) OR (miRNA-155)) OR (microRNA-155)) OR (hsa-mir-155 microRNA)) OR (miR-155-5p)). The specific inclusion criteria inclusion criteria for study eligibility are as follows: (1) clinical study reporting on the roles of miR-155 in AKI patients; (2) experimental research reporting on the effects of miR-155 in AKI and the potential underlying molecular mechanisms; A total of 20 studies, either experimental or clinical, were finally included and further analyzed for this systematic review. The main information from each study, including article information, research object, types of injury, expression of miR-155, associated genes/pathways, and the main findings were collected and extracted by using a specific data collection table. Table 1 summarizes the characteristics of twenty included studies.

**TABLE 1:** The main findings of the eligible studies reported the roles of miR-155 in AKI.

Study/references	Research object	Types of injury	Expression of miR-155	Associated genes/pathways	Main findings
Saikumar et al., 2012[10]	Patients and rats	Ischemia/reperfusion or gentamicin-induced kidney injury	Upregulation	NA	MiR-155 could serve as a biomarker for detection of acute kidney injury
Pellegrini et al., 2014[13]	Mice	Cisplatin-induced nephrotoxicity injury (20 mg/kg, Sigma)	Downregulation	Modulating c-Fos	The inhibition of miR-155 can affect the development of cisplatin-induced kidney toxicity by upregulating c-Fos
Aelst et al., 2016 [20]	Patients	Acute rejection-induced renal injury	Upregulation	NA	MiR-155 could distinguish between rejecting and nonrejecting samples
Millan et al., 2017[14]	Patients	Acute rejection-induced renal injury	Upregulation	NA	Monitoring urinary miR-155-5p provides help in the prognosis of acute rejection
Zhou et al., 2017b[15]	Mice	MTB-induced AKI	Upregulation	NA	MiR-155 induced renal injury by regulating the expression of TNF-α, IL-17 and INF-γ
Glineron et al., 2018[18]	Rats	Puromycin-induced kidney injury (glomerulus, 20/10 mg/kg, i.p.)	Upregulation	NA	Urinary miR-155-5p could be used as a novel marker for monitoring preclinical puromycin-induced kidney injury
Chen et al., 2018[19]	Mice	LPS-induced AKI	Upregulation	Downregulating TNF-a and IL-6 and upregulating SOCS1 and STAT1	MiR-155 inhibitor alleviated LPS-induced kidney injury by suppressing TNF-a and IL-6 in kidney tissue and by increasing the expression of SOCS1 and STAT1
Du et al., 2019[16]	Mice and HK2 cells	Sepsis-induced AKI	Upregulation	Targeting Bcl2	MiR-155 induced tubular cell apoptosis by promoting Bcl2
Zhang et al., 2019[7]	Rats and NRK- 52E cell	Renal ischemia-reperfusion injury	Upregulation	Downregulating TCF4/Wnt/β-catenin pathway	MiR-155 exacerbated AKI by suppressing the TCF4/Wnt/β -catenin signaling pathway
Wang et al., 2020b[8]	Patients and HK-2 cells	Sepsis-induced AKI	Upregulation	NA	MiR-155 inhibition ameliorated Sepsis-induced AKI.
Lu et al., 2020[9]	HK2 cells and transgenic mice	LPS-induced AKI	Upregulation	Downregulating SIRT1	The inhibition of miR-155 alleviated LPS induced renal tubular epithelial cell damage by downregulating SIRT1
Yin et al., 2022[1]	Mice and HK-2 cells	Cisplatin-induced nephrotoxicity injury (18 mg/kg, Sigma)	Upregulation	Downregulating TRF1 and CDK12	MiR-155 deficiency attenuated cisplatin-induced AKI by inhibiting TECs apoptosis, genome instability, and telomeric dysfunction through increasing expression of TRF1 and CDK12
Zhao et al., 2022b[2]	Mice and HK-2 cells	Acute renal allograft injury	Upregulation	upregulating ERRFI1	The inhibition of miR-155-5p attenuatesd acute renal allograft injury through upregulating ERRFI1
Wang et al., 2022b[3]	Mice and HK-2 and TCMK-1 cells	Sepsis-induced acute kidney injury	Upregulation	Downregulating WWC1	MiR-155-5p promoted sepsis-induced AKI progression by downregulation WWC1
Zhang et al., 2022 [4]	Mice	Renal ischemia-reperfusion injury	Upregulation	Downregulating SOCS-1	MiR-155 in macrophage-derived exosomes promoted tubular injury by inhibiting SOCS-1 expression in ischemia-induced AKI.
Petejova et al., 2022[12]	Patients	Vancomycin-induced AKI	Upregulation	NA	miR-155-5p positively correlated with renal injury markers in the vancomycin-induced AKI.
Janosevic et al., 2023[5]	Human kidney tissues	ATN or AIN	Upregulation	NA	MiR-155 expression was upregulated in kidney tissues of AKI patients
You and Kuang 2023[6]	HK-2 cells	Sepsis-induced AKI	Upregulation	Downregulating PDE7A	MiR-155-5p promoted LPS-induced HK-2 cell injury by inhibiting PDE7A expression
Fan et al., 2023[11]	Patients	Sepsis-induced AKI	Upregulation	NA	MiR-155 can serve as a novel diagnostic and prognosis markers in sepsis-induced AKI.
Chen et al., 2024a[17]	Mice and HK2 cells	Ischemia/reperfusion kidney injury	Upregulation	NA	The inhibition of miR-155-3p alleviated renal injury by inhibiting tubular pyroptosis

Note: TRF1 telomeric repeat binding factor 1, CDK12 cyclin-dependent kinase 12, AKI, acute kidney injury, ERRFI1 ERBB, receptor feedback inhibitor 1, WWC1 WW, and C2 domain-containing 1, PDE7A phosphodiesterase 7A, TCF4 transcription factor 4, SIRT1 silent information regulator sirtuin 1, LPS, lipopolysaccharide; MTB, *mycobacterium tuberculosis*; TNF-α, tumor necrosis factor-α; IL-17, interleukin-17, INF-γ, interferon-γ, Bcl2 B-cell lymphoma-2.

Of the included studies, six were clinical trials, and 14 were animal or cell-based studies. All the clinical trials suggested that the expressions of miR-155 were upregulated in the urine, bloods and renal tissue of patients with AKI. Among animal or cell-based studies, 13 showed elevated miR-155 expression, while only 1 showed decreased miR-155 expression. Therefore, miR-155 may act as a protective action in AKI. However, the discordance between these studies indicate that a large series of studies are needed to confirm the role of miR-155 in AKI.

## The roles of miR-155 in the progress of AKI

### Clinical implications of miR-155 in AKI

Among the 20 included studies, six clinical trials evaluated the clinical significance of miR-155 in AKI. Janosevic et al. (Janosevic et al., 2023) demonstrated that miR-155 was significantly upregulated in kidney tissues of patients with the diagnoses of AKI induced by acute interstitial nephritis (AIN) or acute tubular necrosis (ATN). Fan et al. (Fan et al., 2023) collected the blood samples of patients with septic AKI and examined the expression of serum miR-155 by real-time PCR. Meanwhile, the authors evaluated the early diagnosis of miR-155 for septic AKI by drawing the receiver operating characteristic (ROC) curves (Fan et al., 2023). The results showed that the expression of miR-155 in the blood samples of patients with septic AKI was dramatically higher than that in patients with septic non-AKI(Fan et al., 2023). Moreover, miR-155 expression level increased with the aggravation of renal function impairment (Fan et al., 2023). At the cutoff value of 2.37 for miR-155,the optimal sensitivity and specificity for diagnosing septic AKI were 91.12% (95% CI: 80.41%–95.07%), and 4.52% (95% CI: 71.74%–89.36%) respectively (Fan et al., 2023). To evaluate the role of serum miR-155 in the prognosis of septic AKI, the authors divided overall patients into survival group and death group and found that the level of miR-155 in the death group was increased (Fan et al., 2023). Then, all septic AKI patients were divided into low expression group and high expression (n = 42) according to the median of miR-155 (1.81) and found that patients in the low expression group survive longer than the high expression group (Log Rank = 21.78, P < 0.001) (Fan et al., 2023). Similarly, Wang Y. et al. (2020) found that miR-155 expression was significantly elevated in plasma of patients with septic acute lung injury. In addition, its expression level was positively correlated with lung function and could be used as a potential biomarker for early diagnosis of acute lung injury (Wang Z. F. et al., 2020). These results illustrated that serum miR-155 not only served as a diagnostic biomarker for septic AKI but also has an important value in the prognosis assessment of septic patients with AKI. In contrast to this, Saikumar et al. (2012) found that the expression of level of miR-155 was decreased 1.5 fold in patients with ischemic or septic AKI (n = 22) as compared to healthy volunteers (n = 25) through investigating their expression profile in urine samples. However, this study did not measure the expression level of miR-155 in blood samples of AKI. Further study on the opposite result is necessary.

As is well known, nephrotoxicity is one of the adverse effects of vancomycin. Petejova et al. (2022) tracked blood miR-155-5p in patients with sepsis treated with vancomycin and found that circulating miR-155-5p positively correlated with NGAL and creatinine, the important markers of kidney damage. This suggests that miR-155-5p may be used as diagnostic or therapeutic tool in AKI induced by vancomycin. It was reported that post-liver transplantation, the expression of miR-155-5p was higher in T cell-mediated acute rejection and subclinical rejection patients than in non-rejectors, and monitoring miR-155-5p plasmatic expression could serve as a prognostic biomarker for acute and subclinical rejection in liver transplantation recipients (Millan et al., 2019). However, whether miR-155-5p serves a role in kidney graft rejection remains unknown. Recently, Millan et al. (2017) reported that eight of the 80 patients with kidney transplantation experienced acute rejection, and the rejectors showed a significant gradual upregulation of urinary miR-155-5p expression. In addition, the level of miR-155-5p miR-155-5p. Furthermore, miR-155-5p expression level positively correlates with glomerular filtration rate (Millan et al., 2017). The ROC curve analysis demonstrated the excellent capacity of miR-155-5p for predicting acute rejection (Millan et al., 2017). Therefore, an early monitoring of monitoring of urinary miR-155-5p could aid in predicting the outcome in patients with renal transplantation. This was corroborated by Van Aelst et al. (2016), and is also consistent with the results obtained in this study. However, prospective trials with large samples are still required to validate the clinical usefulness of miR-155-5p.

### Protective properties of miR-155 in AKI reported in experimental studies

#### Cisplatin-induced AKI

Cisplatin, a class of cytotoxic agents, has been widely used for treating various solid tumors, including lung cancer, prostate cancer, and breast cancer (Wang et al., 2023; Hu et al., 2021; Altintop et al., 2022). Despite its antineoplastic efficacy, clinical use of cisplatin causes some side effects, such as neurotoxicity, ototoxicity and nephrotoxicity (Tan and Vlajkovic, 2023; Perse, 2021; Karayay et al., 2023). The pathogenesis of cisplatin-induced AKI involves multiple aspects, including direct cytotoxic effects, oxidative stress, inflammation, DNA damage response, and mitochondrial dysfunction (Zhang et al., 2023; Yu et al., 2023; Zhu et al., 2015). As reported, miRNAs play a critical role in cisplatin-induced AKI by regulating key pathological processes. For example, miR-6805-5p and miR-195a promote tubular cell apoptosis and inflammation by targeting anti-apoptotic genes and pro-inflammatory pathways (Yuan et al., 2024; Torso et al., 2023). Conversely, miR-146a-5p and 140-5p protect against renal damage by modulating fibrosis and oxidative stress (Wu et al., 2020; Liao et al., 2017). These miRNAs not only serve as potential biomarkers for early AKI detection but also represent therapeutic targets to mitigate cisplatin nephrotoxicity. Besides, miRNAs play crucial roles in both apoptosis and inflammation. MiRNAs can target pro-apoptotic or anti-apoptotic genes, thereby influencing the sensitivity of cells to apoptosis (Sun et al., 2023). In addition, miRNAs can also regulate inflammation by targeting genes involved in inflammatory signaling pathways (Tahamtan et al., 2023). Li et al. (2024a) reported that lipopolysaccharide (LPS)-exposed HK-2 cells showed increased expression of MiR-16-5p, Bax and caspase-3 and decreased expression of B-cell lymphoma-2 (Bcl-2), and significantly promoted cell apoptosis. Nevertheless, miR-16-5p inhibitor significantly attenuated this effect (Li et al., 2024a). Therefore, inhibition of miR-16-5p can significantly ameliorated sepsis-associated AKI by suppressing apoptosis through targeting apoptosis-related genes. Tubulointerstitial inflammation is a characteristic of acute kidney injury (Zhou Z. Q. et al., 2017). SOCS-1 (suppressor of cytokine signaling) is a negative regulator of NF-κB pathway (El-Sahar et al., 2021). Lv et al. (2020) found that miR-19b-3p was most notably increased in a LPS-induced AKI. Further study demonstrated that miR-19b-3p inhibition suppressed LPS-induced AKI by inhibiting tubulointerstitial inflammation through effecting SOCS-1/NF-κB signaling pathway (Lv et al., 2020). Li et al. (2017) found that miR-155 deficiency attenuated liver ischemia-reperfusion injury by regulating inflammatory response. However, it is unclear whether miR-155 plays a role in cisplatin-induced AKI. Recently, Yin et al. (2022) reported that the expression of miR-155 was upregulated and the expression of cyclin-dependent kinase 12 (CDK12) and telomeric repeat binding factor 1 (TRF1) were downregulated in cisplatin (18 mg/kg, Sigma)-induced AKI. Moreover, knockdown of miR-155 significantly ameliorated cisplatin-induced AKI and improved renal function (Yin et al., 2022). Additionally, inhibition of miR-155 attenuated tubular epithelial cells apoptosis and DNA damage (Yin et al., 2022). Meanwhile, miR-155 knockdown can partially reverse the decreased expression of CDK12 and TRF1 (Yin et al., 2022). These results suggested that miR-155 deficiency significantly attenuated pathological damage in cisplatin-induced AKI by inhibition of tubular epithelial cells apoptosis and telomeric dysfunction through increasing expression of CDK12 and TRF1. However, Pellegrini et al. (2014) demonstrated that miR-155 knockdown significantly promoted cisplatin (20 mg/kg, single)-induced AKI by increasing apoptosis through upregulating c-Fos expression. In addition, Glineur et al. (2018) established a cisplatin (2.5 mg/kg, intraperitoneal (i.p.)-induced AKI rat model and demonstrated that cisplatin induced kidney injury but not induced the expression of miR-155. The causes for the opposite results needed to be further explored. Also, the authors established a puromycin (20 mg/kg, i.p.) -induced AKI model and found that the expression of miR-155-5p significantly increased in the urine of rats after puromycin treatment (Glineur et al., 2018). However, the specific biological mechanism of miR-155-5p on puromycin-induced AKI has not yet been studied.

### Ischemia-reperfusion-induced AKI

Acute renal ischemia–reperfusion injury (RIRI) is characterized by infiltration of immune cells and tubule injury after the reestablishment of the blood supply (Bejoy et al., 2022; Gerhardt et al., 2023). RIRI typically occurs after organ transplantation and infarction. The proximal tubule is the mainstay of RIRI and it has been described as the most common cause of AKI (Perazella and Rosner, 2022). SOCS-1, an inflammation suppressor, normally functions as a negative modulator of NF-κB signaling pathways and has been shown to play essential roles in acute liver injury, cardiac injury and AKI (Bai and Chen, 2023; Ma et al., 2013). Wang Z. et al. (2022) reported that SOCS-1 expression was decreased in hepatic ischemia-reperfusion injury and SOCS-1 upregulation ameliorated hepatic ischemia-reperfusion injury by inhibiting NF-κB signaling pathways. Additionally, miR-155 has been reported to accelerate liver injury by promoting production of inflammatory cytokines through inhibiting the expression of SOCS-1 (Wang et al., 2018). However, it is unknown whether miR-155 play an important role in RIRI by regulating the expression of SOCS-1. Zhang et al. (2022) demonstrated that the expression level of miR-155, BUN and SCr were increased and SOCS-1 expression was decreased in RIRI. Notably, miR-155 inhibition significantly ameliorated tubular injury and reversed the regulation of SCr and BUN (Zhang et al., 2022). Also, the decreased expression of SOCS-1 was reversed by the inhibition of miR-155 (Zhang et al., 2022). Therefore, miR-155 inhibition ameliorated RIRI-induced AKI by suppressing tubular injury through upregulating SOCS-1 expression.

Recently, miR-155-5p upregulation has been shown to induce cell apoptosis by activating Wnt/β-catenin signal pathway through targeting TCF4 (Prossomariti et al., 2018). In addition, it was reported that the inhibition of miR-144-5p promoted RIRI-induced AKI by activating Wnt/β-catenin signal (Xu Y. et al., 2022). Zhang et al. (2019) demonstrated that miR-155 and β-catenin expression were increased while TCF4 expression was reduced in RIRI-induced AKI. Furthermore, miR-155 mimic promoted RIRI-induced apoptosis and inhibited the expression of TCF4, a positive regulator of Wnt/β-catenin signaling pathway (Zhang et al., 2019). Moreover, TCF4 overexpression restored miR-155-mediated RIRI-induced apoptosis of renal tubular epithelial cells (Zhang et al., 2019). These results were consistent with previous studies showing that the Wnt/β-catenin signaling pathway plays a key role in the protection of AKI (Correa-Costa et al., 2012; Terada et al., 2003). Accordingly, these findings suggested that miR-155 promoted RIRI-induced AKI by enhancing cell apoptosis through the inactivation of the TCF4/Wnt/β-catenin pathway.

Pyroptosis, a type of programmed cell death, is also considered as one of the main forms of cell death in AKI (Fu et al., 2023). It was reported that pyroptosis amplified renal injury through both aggravation of inflammation and direct cell death by pro-inflammatory cytokines. However, the regulation of pyroptosis was complex. Recently, gap junction, a type of channel for information exchange, has been shown to be involved in the regulation of pyroptosis in various physiological processes (Gilleron et al., 2018). Li et al. (2019) demonstrated that connexin43 (Cx43), a gap junction protein regulating cell growth and apoptosis, significantly attenuated pyroptosis of human umbilical vein endothelial cells by decreasing the level of active caspase-1. However, Cx43 silencing significantly promoted pyroptosis by increasing the expression of caspase-1 (Li et al., 2019). Bian et al. (Bian et al., 2017) reported that miRNA-1 downregulation might protect the heart from ischemia-reperfusion injury by preventing the decrease and redistribution of Cx43. More recently, Chen L. et al. (2024) treated with hypoxia reoxygenation to mimic RIRI and found that hypoxia reoxygenation could lead to pyroptosis of NRK-52E and HK-2 cells and induced the expression of miR-155-3p and production of connexin32. Furthermore, Cx32 inhibition significantly reduced the pyroptosis of NRK-52E and HK-2 cells and suppressed the expression of miR-155-3p after hypoxia reoxygenation (Chen L. et al., 2024). Moreover, this effect could be reversed by miR-155-3p mimic (Chen Y. et al., 2024). These findings suggested that inhibition of Cx32 could alleviate RIRI-induced AKI by inhibiting pyroptosis through reducing the level of miR155-3p.

Live-donor kidney transplantation (LDKT) is one of the therapeutic options for eligible patients with advanced chronic renal disease (Voora and Adey, 2019). Unfortunately, ischemic AKI is often experienced during renal allograft, which may eventually result in renal function loss, and the possible underlying mechanism ischemia/reperfusion injury (Chen Y. et al., 2024). However, the occurrence of ischemic AKI cannot be anticipated after renal allograft. Therefore, new therapeutic targets for acute renal allograft injury are desperately needed. As earlier reported, regulation of miRNA is very beneficial for mitigating ischemia/reperfusion injury during renal allograft, such as miR-20a-5p. Shi et al. (2023) reported that miR-20a-5p significantly alleviated ischemia/reperfusion injury via regulating ferroptosis after kidney transplantation. ERBB receptor feedback inhibitor 1 (ERRFI1) has been shown to negatively regulate the production of inflammatory mediators and be a target of miRNAs. Guo et al. (Guo et al., 2019) demonstrated that miR-2355-5p significantly inhibited nucleus pulposus cells proliferation and inflammation by negatively regulating ERFFI1. Wang et al. (2019) found that miR-126 alleviated ischemic reperfusion injury by attenuating oxidative stress and apoptosis through regulating ERFFI1. Recently, miR-155-5p was enhanced, and ERRFI1 was suppressed in mice after renal allograft and hypoxia/reoxygenation-treated HK-2 cells (Zhao J. et al., 2022). Furthermore, depletingmiR-155-5p significantly reduced serum inflammation and attenuated oxidative stress, apoptosis, and renal tubular injury in mice with acute renal allograft injury (Zhao J. et al., 2022). Similarly, miR-155-5p knockdown repressed apoptosis and facilitated proliferation of hypoxia/reoxygenation-treated HK-2 cells (Zhao S. et al., 2022). Moreover, downregulating miR-155-5p significantly inceased the expression level of miR-155-5p (Zhao S. et al., 2022). Interestingly, knocking down ERRFI1 reversed the effects of inhibition of miR-155-5p on hypoxia/reoxygenation-treated HK-2 cells apoptosis and proliferation, as well as on acute renal allograft injury (Zhao J. et al., 2022). Taken together, silencing miR-155-5p could attenuate acute renal allograft injury by enhancing the expression of ERRFI1, which provides a way to control ischemic AKI.

### Sepsis-associated AKI

Sepsis can lead to a systemic inflammatory response syndrome (Ling et al., 2023). The common clinical symptoms of this disease include tachycardia, tachypnea, shortness of breath, etc., (Eisenberg and Balamuth, 2022). Typically, severe sepsis is accompanied by hypotension, hypoperfusion and dysfunction of at least one organ (Zampieri et al., 2023). It was reported that patients with severe sepsis have a 60% probability of developing AKI (Zafrani et al., 2016). The pathophysiological mechanisms of sepsis-induced AKI are involved in cell injury, oxidative stress, apoptosis, and mitochondrial dysfunction (Xiao et al., 2023; Joffre and Hellman, 2021). STAT1 has been reported to be a key signaling molecule for growth factors and a variety of cytokines to transmit signals in cells and be also involved in the pathological processes of apoptosis and immune response (Li S. et al., 2021). In addition, IL-6 and TNF-α are also involved in multiple inflammatory and autoimmune diseases, including lupus nephritis, acute lung and AKI (Wang M. et al., 2020; Chen et al., 2023). Xie et al. (2020). reported that miR-128-3p significantly promoted the progression of AKI by reducing G1 arrest and apoptosis through targeting STAT1. Recently, Chen et al. (2018) used the LPS model of sepsis to explore the role of miR-155 on sepsis-induced AKI and demonstrated that the increased expression of miRNA-155 in LPS-treated group was eight times higher than in non-treated control group. Furthermore, the expression levels of SOCS-1, STAT1, IL-6 and TNF-α were elevated in the kidney tissues of LPS model (Chen et al., 2018). Interestingly, miRNA-155 inhibitor suppressed the expression of STAT1, IL-6, and TNF-α, but promoted the expression of SOCS-1 (Chen et al., 2018). Moreover, necrosis of the glomerulus, inflammatory cell infiltration and tubular epithelial swelling were clearly observed in LPS treated group (Chen et al., 2018). Importantly, these pathological concerns of the kidneys were remarkably reduced by miRNA-155 inhibition (Chen et al., 2018). Consistently, Wang M. et al. (2020) also found that miR-155 inhibition could suppress sepsis-induced AKI by regulating inflammation. These results indicated that miR-155 inhibitor alleviated sepsis-induced AKI by remarkably suppressing STAT1, TNF-α and IL-6 and by significantly increasing the expression of SOCS-1. WW and C2 domain-containing 1 (WWC1) is the target gene of miR-155 (Giri et al., 2024). WWC1 overexpression apparently reduced the productions of inflammatory cytokines, such as TNF-α, IL-6, and IL-1β. WWC1 has been shown to play a crucial role in brain injury (Gusareva et al., 2018). Also, during the progression of sepsis-induced AKI, WWC1 is regarded as an important suppressor of inflammatory cytokines. MiR-155-5p promoted sepsis-induced AKI progression by inhibiting WWC1 expression (Wang and Cao, 2022). In addition, SIRT1, Bcl2 and PDE7A were confirmed to be the target genes of miR-155, which promoted or inhibited liver injury, lung injury and AKI by regulating inflammation or cell apoptosis (Yang et al., 2018; Tuerdi et al., 2018; You and Kuang, 2023). It has been reported that miR-155 aggravated sepsis-induced AKI by inducing tubular cell apoptosis through modulating the expression levels of these genes (You and Kuang, 2023; Du J et al., 2019; Lu et al., 2020). In summary, these findings suggested that miR-155 promoted the progression of sepsis-induced AKI by targeting various proteins or signaling pathways, and miR-155 inhibition might be an effective method of treatment for sepsis-induced AKI.

### *Mycobacterium tuberculosis* infection associated AKI

Tuberculosis is still a major public health concern and is expected to present greater challenges in the Western Pacific and South-East Asia regions (Tong et al., 2017). It has been shown that among extra-pulmonary tuberculosis, 6%-8% of cases involved the genitourinary tract (Daher et al., 2013). *Mycobacterium tuberculosis* spreads to kidney and leads to extensive destructive caseous lesions and ulceration, which induces AKI (Yadav et al., 2017). However, owing to poor culture techniques, early diagnostic efficiency of *mycobacterium tuberculosis* infection-induced AKI is usually unsatisfactory. The common treatment methods of AKI include renal replacement therapy, cell-based therapies and correction of uremia-associated factors (Togel and Westenfelder, 2012). Recently, the early secreted antigenic target-6 (ESAT-6), secreted by *mycobacterium tuberculosis*, was found to be a potential therapeutic target of AKI. Gao et al. (2015) reported that the pathological score of renal injury was gradually increased and BUN and Scr increased gradually in the experimental group after ESAT-6 infection. This indicated that ESAT-6 might contribute to the development of AKI. Former studies demonstrated that ESAT-6 expression was strongly and positively related to miR-155 expression (Kumar et al., 2012; Yang et al., 2015). Zhou X. et al. (2017) also found that *mycobacterium tuberculosis* infection could induce renal injury and miR-155 overexpression aggravated renal injury induced by *mycobacterium tuberculosis* infection. Conversely, miR-155 inhibition suppressed renal injury induced by *mycobacterium tuberculosis* infection (Zhou X. et al., 2017). Further mechanism study reveals that ESAT-6 induced renal injury by enhancing miR-155 expression via the TLR-4/MyD88 signaling pathway (Zhou et al., 2017b). These results suggested that miR-155 might play the promoting role in *mycobacterium tuberculosis* infection-induced AKI.

### Roles of miRNAs and immune cells in the infectious diseases associated with AKI

Infectious diseases, i.e., sepsis, COVID-19, and malaria, have been found to precipitate AKI by directly damaging renal cells and dysregulating immune responses, with pathogens like *Leptospira* and hantavirus exacerbating vascular leakage and tubular ferroptosis (Kuwabara et al., 2022; Li et al., 2024b; Chen and Gan, 2023). Immune cells, including neutrophils, M1-polarized macrophages, Th17 cells, and CD103+ dendritic cells, are confirmed to orchestrate AKI progression, while regulatory miRNAs like miR-155 (SOCS1/STAT3 amplification in M1 macrophages), miR-21 (PDCD4/NF-κB/NLRP3 axis), miR-146a (IRAK1/TRAF6 suppression via AAV9 delivery), and miR-223 (NLRP3/CXCL2 inhibition in liposomes) can modulate inflammation. Therefore, infectious diseases may link to AKI and immune cells/miRNAs regulatory networks, offering precision strategies to mitigate AKI.

### The interactions between miR-155-associated molecules/pathways and AKI

Based on the other relevant studies, miR-155 has been found to involve in the development and progression of AKI and other kidney diseases by interacting with some molecules/pathways. The roles of miR-155 and STAT6 in AKI are distinct but interconnected through inflammatory and fibrotic pathways. Prieto et al. reported that miR-155 upregulation correlated with albuminuria, inflammation, and fibrosis in diabetic kidney disease (Prieto et al., 2023). It suppresses SOCS1 (a negative regulator of JAK/STAT signaling), leading to STAT1/3 activation, cytokine production (e.g., TNF-α, IL-6), and mesangial cell proliferation/migration. In lipopolysaccharide (LPS)-induced AKI, miR-155 inhibition reduced STAT3 phosphorylation, inflammation, and apoptosis, highlighting its role in septic AKI (Ren et al., 2017). miR-155 directly targeted SOCS1, forming a reciprocal loop where miR-155 promotes inflammation by SOCS1-mediated inhibition of JAK/STAT signaling. This loop exacerbates renal damage and sepsis. STAT6 is a key gene on the pathway of JAK/STAT signaling. Previous study showed that STAT6 exerted an important function in myeloid fibroblasts activation of the kidneys (Jiao et al., 2021). Other studies demonstrated that STAT6 phosphorylation could upregulate miRNA-155 expression in dozens of diseases (Chen et al., 2019). Therefore, targeting miR-155 and STAT6 could disrupt these parallel pathways to mitigate inflammation and fibrosis in diverse kidney pathologies.

Other molecules and cascades, like chromosome ten (PTEN), Jumonji domain-containing protein-3 (JMJD3), and cGAS-STING, have also been found to play a pivotal role in the development and progression of AKI and other renal diseases (Pasca et al., 2020). Guo et al. (2020) reported that dihydromyricetin enhanced cellular autophagy and improved renal interstitial fibrosis by modulating both the miR-155-5p/PTEN and PI3K/AKT/mTOR signaling cascades in diabetic nephropathy. A previous study demonstrated that the observed elevation in H3K27 dimethylation during renal fibrosis development coincided with significantly heightened JMJD3 expression in renal parenchyma, which might be mediated by regulating with the M2 macrophage polarization in the obstructed kidney (An et al., 2023). The findings from Jiao and colleagues established cGAS-STING activation as a functional axis bridging innate immune responses with macrophage-driven inflammatory processes in progressive renal fibrosis (Jiao et al., 2025). Since the interactions between various micro-RNAs and the abovementioned molecules have been identified in the involvement of multiple kidney diseases. Therefore, miR-155 may also play an essential role in the pathomechanisms of AKI.

## Conclusions and prospects

**FIGURE 2:**
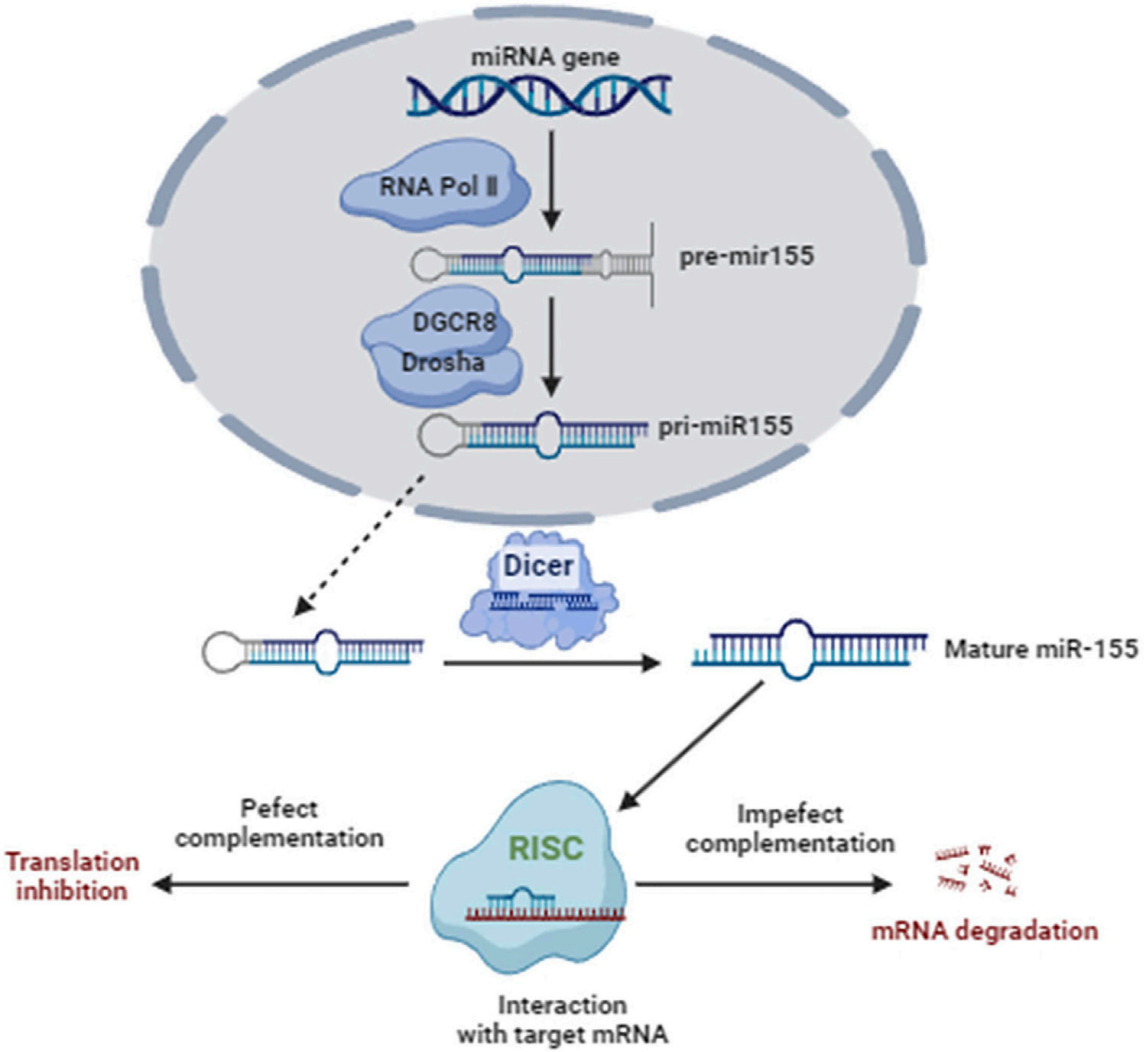
The molecular mechanisms of miR-155 on AKI.

Based on this review, miR-155 is involved in cell apoptosis and proliferation and is also strongly correlated with the pathophysiological development of AKI. Based on the studies included in this review, the inhibition of miR-155 has a reno-protective effect in drug- or substance-induced AKI. The molecular mechanisms of miR-155 on AKI are illustrated in Figure 2. As listed in Table 1, among the twenty studies included, only six studies reported the clinical applications of miR-155 in AKI, which limited its broad prospects. Detection of miR-155 may be useful in the early diagnosis of AKI due to various causes and is helpful in predicting the prognosis of AKI due to various causes. Currently, a miRNA nanocarrier system that has already been developed could offer a novel approach for treating AKI. Emerging miR-155 nanocarrier systems for AKI have been established, including pH-responsive lipid-polymer hybrids, galectin-1-targeted mesoporous silica, and CD133-engineered exosome mimetics, synergizing with mitochondrial rescue agents (e.g., MitoQ nanoparticles), epigenetic modulators (HDAC6 inhibitors), and CAR-macrophage immunotherapies to address multifactorial pathology.

MiR-155 exerts a promoting function in multiple types of AKI by regulating multiple proteins or signaling pathways, such as SOCS-1, ERRFI1, SOCS-1, TRF1, CDK12, and TCF4/Wnt/β-catenin pathway. Therefore, drugs or biological compounds targeted by miR-155 and its pathways may recover the process of multiple types of AKI by regulating apoptosis, inflammation and pyroptosis. However, the clinical applications of these drugs or biological compounds remain several challenges. We posit that a prospective, large-scale clinical trial will play a key role in promoting the translation of these findings into clinical practice for treating AKI.
